# Molecular diversity of *Bulinus* species in Madziwa area, Shamva district in Zimbabwe: implications for urogenital schistosomiasis transmission

**DOI:** 10.1186/s13071-020-3881-1

**Published:** 2020-01-10

**Authors:** Masceline Jenipher Mutsaka-Makuvaza, Xiao-Nong Zhou, Cremance Tshuma, Eniola Abe, Justen Manasa, Tawanda Manyangadze, Fiona Allan, Nyasha Chinómbe, Bonnie Webster, Nicholas Midzi

**Affiliations:** 1 Department of Medical Microbiology, College of Health Sciences, University of Zimbabwe, P.O. Box A178, Avondale, Harare, Zimbabwe; 2 National Institute of Health Research, Ministry of Health and Child Care, P.O. Box CY573, Causeway, Harare, Zimbabwe; 3 National Institute of Parasitic Diseases, Chinese Centre for Disease Control and Prevention, Shanghai, 200025 China; 4 Mashonaland Central Provincial Health Office, Ministry of Health and Child Care, Bindura, Mashonaland Central Zimbabwe; 5 Geography Department, Faculty of Science, Bindura University of Science Education, Bag 1020, Bindura, Zimbabwe; 6 School of Nursing and Public Health, Department of Public Health Medicine University of KwaZulu-Natal, Durban, South Africa; 7 Wolfson Wellcome Biomedical Laboratories, Department of Life Sciences, Natural History 14 Museum, Cromwell Road, London, SW7 5BD UK

**Keywords:** *Bulinus globosus*, *Bulinus truncatus*, *Cox*1, Diversity, Phylogenetics, Zimbabwe

## Abstract

**Background:**

*Bulinus* species are freshwater snails that transmit the parasitic trematode *Schistosoma haematobium*. Despite their importance, the diversity of these intermediate host snails and their evolutionary history is still unclear in Zimbabwe. *Bulinus globosus* and *B. truncatus* collected from a urogenital schistosomiasis endemic region in the Madziwa area of Zimbabwe were characterized using molecular methods.

**Methods:**

Malacological survey sites were mapped and snails were collected from water contact sites in four communities in the Madziwa area, Shamva district for a period of one year, at three-month intervals. *Schistosoma haematobium* infections in snails were determined by cercarial shedding and the partial mitochondrial cytochrome *c* oxidase subunit 1 gene (*cox*1) was used to investigate the phylogeny and genetic variability of the *Bulinus* spp. collected.

**Results:**

Among the 1570 *Bulinus* spp. snails collected, 30 (1.9%) *B. globosus* were shedding morphologically identified schistosomes. None of the *B. truncatus* snails were shedding. The mitochondrial *cox*1 data from 166 and 16 samples for *B. globosus* and *B. truncatus*, respectively, showed genetically diverse populations within the two species. Twelve *cox*1 haplotypes were found from the 166 *B. globosus* samples and three from the 16 *B. truncatus* samples with phylogenetic analysis showing that the haplotypes fall into well-supported clusters within their species groups. Both *B. truncatus* and *B. globosus* clustered into two distinct lineages. Overall, significant negative values for both Tajima’s D statistic and the Fu’s Fs statistic were observed for *B. globosus* and *B. truncatus*.

**Conclusions:**

The study provided new insights into the levels of genetic diversity within *B. globosus* and additional information on *B. truncatus* collected from a small geographical area in Zimbabwe. Low prevalence levels of infection observed in the snails may reflect the low transmission level of urogenital schistosomiasis in the area. Our results contribute towards the understanding of the distribution and population genetic structure of *Bulinus* spp. supporting the mapping of the transmission or risk of transmission of urogenital schistosomiasis, particularly in Zimbabwe.

## Background

Freshwater snails of the genus *Bulinus* act as intermediate hosts for *Schistosoma haematobium*, the human blood fluke that causes the chronic and debilitating disease, urogenital schistosomiasis [1]. *Bulinus* species are extensively distributed throughout Africa, countries bordering the Mediterranean and some areas of the Middle East, but there is a considerable variation in compatibility between the different *Bulinus* species and schistosome parasites in different geographical areas [1, 2]. The host-parasite compatibility is influenced by both biotic and abiotic factors such as temperature, rainfall, water velocity, desiccation, salinity and the genetics of the snail hosts and the parasites [2–7].

Among the *Bulinus* species, *B. globosus* (Morelet, 1866) and *B. truncatus* (Audouin, 1827) are recognized as the most important intermediate hosts for *S. haematobium* and the species are distributed widely in Africa [8]. In Zimbabwe, *B. globosus* is the only intermediate host for *S. haematobium* [6]. The intermediate host snail prefers diverse habitat types and is more abundant in the northeast and southeast parts of the country with a patchy distribution in the southwest, correlating with the distribution of urogenital schistosomiasis in Zimbabwe. *Bulinus truncatus* is more common to the southwest preferring dams and marshy habitats [9].

While both *B. truncatus* and *B. globosus* are hermaphroditic, *B. globosus* preferentially cross-fertilizes if possible whereas *B. truncatus* preferentially self-fertilizes [10, 11]. Both species inhabit diverse habitats such as rivers, irrigation systems, ponds and lakes and are tolerant to differing water levels and seasonal changes, and have the ability to aestivate during dry seasons [8]. Geographical and temporal variability in ecological factors can cause large fluctuations in snail abundance within and between sites, possibly resulting in population extinction and/or recolonization. Selfing and population bottlenecks reduce genetic diversity within a population while increasing genetic differences between isolated populations [12]. Investigating snail population structure will help understand snail-parasite relationships and parasite transmission dynamics [13–19].

Due to the high levels of variation in shell morphology within and among *Bulinus* species, morphological identification can be subjective [8, 20]. Nevertheless, it has been the main method used to characterize *Bulinus* species in Zimbabwe [6–8], except for a few studies that utilized iso and alloenzyme analysis [21, 22] and random amplification of polymorphic nuclear DNA (RAPD) analysis [23]. However, several studies conducted in the region have characterized *Bulinus* populations using the DNA sequence analysis of the internal transcribed spacer (ITS) rDNA regions [18, 19, 24] and the mitochondrial cytochrome *c* oxidase subunit 1 (*cox*1) [13, 14, 17–19], with *cox*1 data being more informative for resolving species level affinities within the genus *Bulinus* [13, 14, 19]. Abe et al. [19] have reported six unique mitochondrial *cox*1 haplotypes for *B. truncatus* in Zimbabwe. However, no information exists regarding *cox*1 diversity for Zimbabwean *B. globosus* in the sequence databases. Here, we use mitochondrial *cox*1 data to identify and characterize *Bulinus* snails sampled from different water contact sites in a urogenital schistosomiasis endemic area, Madziwa in Zimbabwe and discuss the findings in relation to schistosomiasis transmission.

## Methods

### Study area

Natural populations of *Bulinus* species were collected from four rural communities in Mashonaland province, in a district highly endemic for urogenital schistosomiasis, as described in a previous study [25]. The communities are in semiarid areas and they rely on local rivers and streams for most of the household activities including bathing, fishing, swimming, washing, gardening and subsistence farming. There are only a few boreholes that are used by the residents for provision of drinking water but these are located ~ 5 km away. Within the four communities, there are distinct periods of high and low rainfall patterns, with the main rainfall period being from late October to April.

### Sample collection, morphological analysis and patent testing

**Fig. 1:**
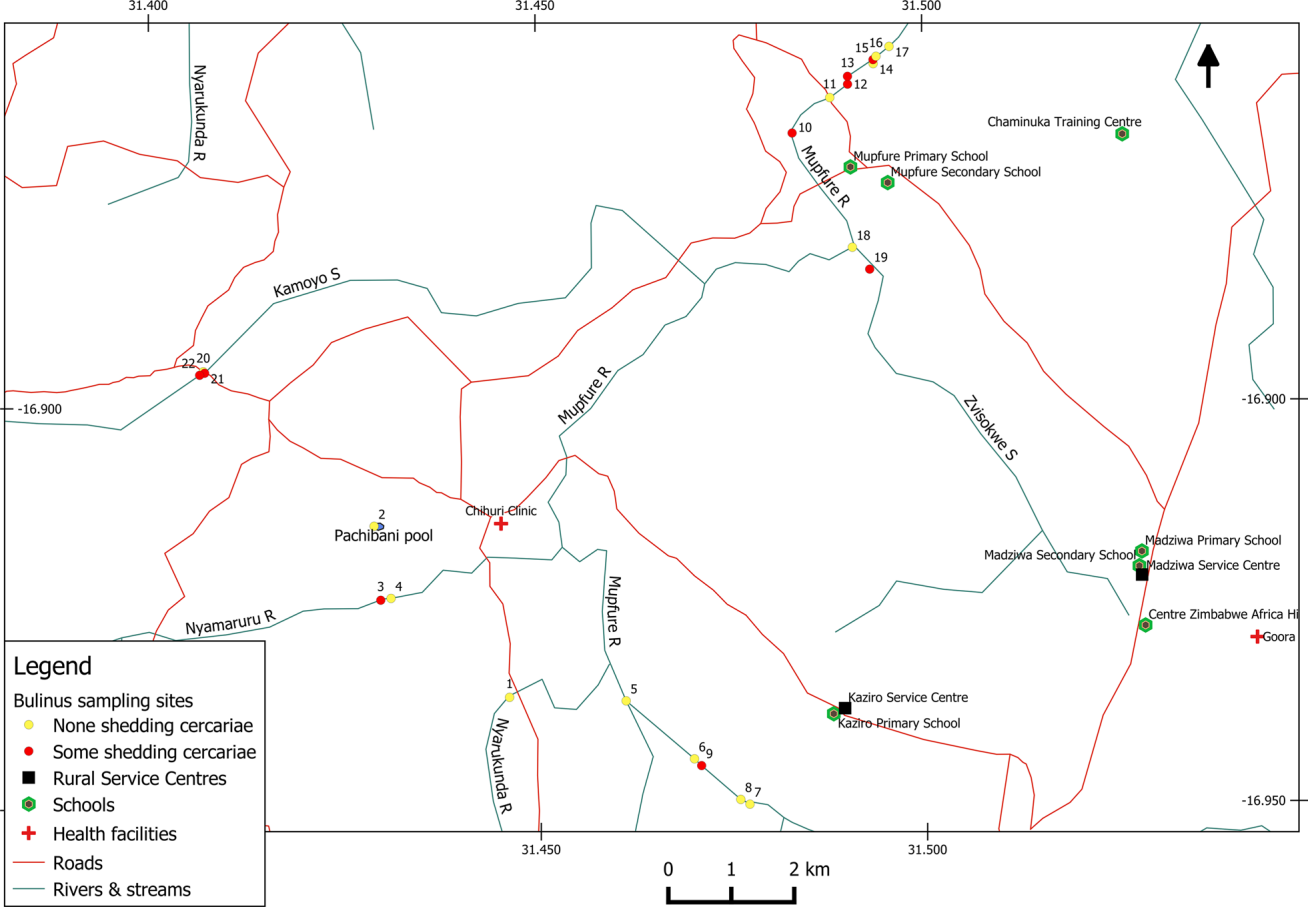
Location of the water contact sites where *Bulinus* snails were collected in Madziwa area, Shamva district, Zimbabwe

This study was part of a larger project investigating the burden of urogenital schistosomiasis in a highly endemic area of Zimbabwe, during which snail survey sites were selected by asking the residents to identify the sites which they frequently used for human water contact activity. The water contact sites are described in Fig. 1. Spatial variation in snail populations was investigated by collecting snails at each water contact point in the communities and all the surveyed sites were mapped using a global positioning system (GPS) (Trimble Navigation Ltd, California, USA). At each water contact site, sampling was performed at four different time points representing the rainy season, post-rainy season, winter and hot-dry season to investigate the temporal effects of season on the snail populations. Snail sampling at all sites was performed using a metal scoop or by handpicking the snails for 30 min at both the main parts of the water body and at the water edges. The snails were transported to the field laboratory where they were identified using shell morphology as described by Brown [8]. Snails with globose, ovate shells of small to medium size, and sinistral with a pseudobranch were grouped as belonging to the genus *Bulinus*. From the snails identified as *Bulinus* spp., *B. globosus* was identified by their truncate columella and microsculpture of nodules with short ridges or corrugations. *Bulinus* snails with a straight or evenly concave or twisted columella with no truncation were identified as *B. truncatus*. Both species were tested for patent parasitic infections by cercarial shedding. The snails were individually placed in flat-bottomed glass vials containing dechlorinated water and exposed to artificial light for a maximum of 4 h [26]. During the 4-hour period, the emergence of cercariae was checked at regular short intervals. Cercarial identification was based on morphology using a binocular microscope as described by Frandsen & Christensen [27]. Bifurcate cercariae were considered to be of mammalian origin. After shedding, the snails were counted and preserved in absolute ethanol for molecular analysis.

### DNA extraction and amplification

Among the collected and morphologically identified snails, a subset of 166 *B. globosus* and all the collected 16 *B. truncatus* were molecularly analysed. Up to eight adult snails were randomly selected per site for molecular analysis [23]. The preserved snails were transported to the Key Schistosomiasis Laboratory, National Institute of Parasitic Diseases, Shanghai, China, for molecular analysis. Snails were removed from ethanol and their soft parts were removed from the snail shell using forceps. For each snail, the soft part was then transferred to a clean Petri dish and immersed in TE buffer (10 mM Tris, 0.1 mM EDTA) pH 7.4 for 1 h. DNA extraction was performed from the head and foot region of the snail tissue using the DNeasy Blood and Tissue Kit (Qiagen, Crawley, UK) following the manufacturer’s instructions. DNA quantification and purity were measured using a Nanodrop ND-1000 spectrophotometer (Nanodrop Technologies Inc., Willington, USA).

### Amplification and sequencing of mitochondrial DNA

The *cox*1 fragment was amplified by PCR, using the BulCox 5 forward (5′-CCT TTA AGA GGN CCT ATT GC-3′) and BulCox 14 reverse (5′-GGA AAT CAG TAM AYA AAA CCA GC-3′) primers previously described by Kane et al. [18] in a C1000TM thermal cycler (Bio-Rad, California, USA). Amplification was carried out in 25 μl PCR reactions containing a premixed master mix (2.5 μl of 20 mM MgCl_2_, 2.5 μl, 5× buffer, 2.5 μl of 20 mM dNTPs, one unit of Taq DNA polymerase), 1 μl (10 pmol) of each forward and reverse primer and 1 μl (10–100 ng) of the DNA template. The PCR conditions were as follows: denaturation at 95 °C for 5 min followed by 45 cycles of 95 °C for 5 min, 45 °C for 30 s and 72 °C for 1 min and a final extension step at 72 °C for 10 min. Negative controls (no template DNA) were included with each set of reactions. PCR products were viewed by 1% gel electrophoresis (Electrophoresis power supplies E455, CONSORT, Turnhout, Belgium) and read with a Molecular Imager, Gel Doc^TM^ XRt Imaging system (Bio-Rad). Amplicons were purified and Sanger sequenced in both forward and reverse directions using the BigDye v3.1 Terminator Cycle Sequencing Kit and read with the automated DNA fragment analyzer ABI-377 (Applied Biosystems, Carlsbad, USA) at Sangon Biotech, Shanghai, China.

### Data analysis

All *cox*1 sequences were manually checked and edited using Sequencher v5.1 (http://www.Genecodes) to remove any ambiguities between forward and reverse strands. The consensus sequences of each sample were aligned in Sequencher v5.1 and any ambiguities between sequences were checked by visualisation of the original sequence chromatograms. Sequences were identified by BLAST searching [28] against the GenBank database.

#### Haplotype analysis

The consensus sequences from all samples were grouped and aligned in MacClade 4.05 and then collapsed together using Collapse v1.2 (http://darwin.uvigo.es/software/collapse.html) to identify samples with identical sequences/haplotypes. Within each site, and also overall, unique sequences and any group of identical sequences represented unique individual haplotypes. A group was defined as a collection of sequences regardless of the site where they were collected. Haplotypes were given a site-haplotype identifier code, consisting of a site number, letter H (haplotype for *B*. *globosus*) or T (haplotype for *B*. *truncatus*) and a number representing the different haplotypes in the area. Overall and in each site, the number of individual *Bulinus* snails presenting the same haplotype was recorded. Haplotype data were submitted to the EMBL/GenBank database under the accession numbers MN397785–MN397822.

#### Phylogenetic analysis

The haplotype data were exported to MEGA 6.0 [29] and aligned using the Clustal W algorithm [30]. Phylogenetic relationships between the haplotypes were inferred using the Neighbor-Joining (NJ), Minimum Evolution (ME) and Maximum Likelihood (ML) methods and the tree topologies were tested using 1000 bootstrap replicates in MEGA 6.0 [29]. The analysis was run using the Tamura-Nei with Gamma distribution nucleotide substitution model, which was the best-fit model for the data, inferred using the model test function in MEGA 6.0 [29, 31, 32]. A sequence for *Biomphalaria glabrata* (GenBank: AY380531) was used as the outgroup. Additional sequences for *Bulinus* spp. (*B. globosus*, *B. africanus* and *B. truncatus*) from Uganda, South Africa, Egypt, Tanzania, Kenya, Senegal, Portugal and Zimbabwe available on GenBank were also included in the analysis (Additional file 1: Table S1). The net nucleotide divergence between the main haplotype groups found within the study was calculated in MEGA 6.0 [29] with the Juke-Cantor model [33].

#### Population genetic analysis

The data were exported into DnaSP v6 [34] to determine the interpopulation and intrapopulation diversity of the *Bulinus* populations analysed. A population was defined as a collection of sequences from the same site or community or overall. Only 16 *B. truncatus* were collected, with not enough representatives per site and so were analysed as a single population. For *B. globosus* the overall diversity, diversity within the sites and also within the communities was measured. Haplotype diversity (h) and nucleotide diversity (π), was calculated using the Juke-Cantor model [33]. The diversification index (*Fst*) between each pair of sites and between *B. globosus* and *B. truncatus* was estimated in DnaSP v6 [34].

#### Test for selection

To investigate if there was significant selection occurring, Tajima’s D [35] and Fu’s Fs statistic neutrality tests for selection were conducted in DnaSP v6. Nucleotide divergence within and between the communities was calculated in DnaSP v6 [34].

## Results

### Morphological identification and infection status of *Bulinus* species

From the collected snails, 1558 were putatively identified as *Bulinus* spp. based on their sinistral, ovate, globose shells. Among these, 1542 were morphologically identified as *B. globosus* and 16 as *B. truncatus*, based on the shape of the columella. Of these, a subset of 166 *B. globosus* and all 16 *B. truncatus* were molecularly characterized with a 100% match between the morphological and molecular species identification.

Among the *B. globosus* collected and tested for patent infections, 30/1542 (1.9%) were shedding schistosome cercariae (Table 1). The breakdown of infected snails by time point was as follows: 18 snails at baseline (end of February 2018; rainy season) and 12 snails at 6 months follow-up (September 2018; summer). No snails were shedding at 3 (winter season), 9 (early rainy season), or 12 (rainy season) months follow-up. None of the *B. truncatus* were found to be infected.

**Table 1:** Demographic information of the *Bulinus* samples analysed in Madziwa, Zimbabwe

Community (prevalence of USCH%)^a^	Water body	Latitude	Longitude	Collection site ID	No. of snails collected	No. of *B. globosus* collected	*B. globosus* shedding cercariae *n* (%)	No. of *B. truncatus* collected^b^	Haplotypes
Chihuri (22.8)	Nyarukunda R	16°56.185′S	31°26.762′E	1	117	117	0 (0)	0	H1, H3
	Pachibani pool	16°54.899′S	31°25.721′E	2	20	20	0 (0)	0	H1, H2, H7
	Nyamaruru R	16°55.452′S	31°25.768′E	3	64	63	1 (1.6)	1	H1, H2, T1
	Nyamaruru R	16°55.439′S	31°25.848′E	4	81	81	0 (0)	0	H2, H3, H6, H12
Sub-total					282	281	1 (0.4)	1	
Kaziro (12.3)	Mupfure R	16°56.997′S	31°28.622′E	7	22	20	0 (0)	2	H1, T2
	Mupfure R	16°56.961′S	31°28.551′E	8	52	50	0 (0)	2	H1, T2, H3
	Mupfure R	16°56.706′S	31°28.249′E	9	35	35	1 (2.9)	0	H1, H3, H5, H7
Sub-total					109	105	1 (1.0)	4	
Mupfure (17.7)	Mupfure R	16°56.655′S	31°28.194′E	5	42	41	0 (0)	1	H1, H5, H8, T2
	Mupfure R	16°56.218′S	31°27.667′E	6	112	112	0 (0)	0	H1, H3, H5, H8, H11
	Mupfure R	16°51.986′S	31°28.347′E	10	208	208	2 (1.0)	0	H1, H3, H6
	Mupfure R	16°51.723′S	31°29.283′E	11	45	45	0 (0)	0	H1, H2, H3
	Mupfure R	16°51.624′S	31°29.422′E	12	26	25	1 (4.0)	1	H1, H4, T3
	Mupfure R	16°51.565′S	31°29.422′E	13	57	57	5 (8.8)	0	H1, H2, H3, H9
	Mupfure R	16°51.472′S	31°29.622′E	14	85	85	0 (0)	0	H1, H3
	Mupfure R	16°51.442′S	31°29.621′E	15	135	135	11 (8.1)	0	H1, H2, H4
	Mupfure R	16°51.417′S	31°29.644′E	16	9	9	0 (0)	0	H1, H2, H7, H10
	Mupfure R	16°51.344′S	31°29.746′E	17	11	10	0 (0)	1	H1, H2, T1
	Zvisokwe S	16°52.842′S	31°29.450′E	18	75	75	0 (0)	0	H1, H4
	Zvisokwe S	16°53.007′S	31°29.583′E	19	220	220	5 (2.3)	0	H1, H4
Sub-total					1025	1022	24 (2.3)	3	
Nduna (12.9)	Kamoyo S	16°53.735′S	31°24.405′E	20	20	16	0 (0)	4	H1, T1
	Kamoyo S	16°53.746′S	31°24.415′E	21	58	54	2 (3.7)	4	H1, T1
	Kamoyo S	16°53.761′S	31°24.376′E	22	64	64	2 (3.1)	0	H1
Sub-total					142	134	4 (3.0)	8	
Total of all sites					1558	1542	30 (1.9)	16	

^a^Prevalence of the disease in women and preschool aged children in the communities studied [25]

^b^None of the *B. truncatus* snails collected was infected

*Notes*: Details for the sites and locations where the snail samples were collected, the times of the collections, the total numbers of snails collected and those infected with schistosomes. The haplotypes column shows the different haplotypes that were observed at each site

*Abbreviations*: R, river; S, stream; H, haplotype of *B. globosus*; T, haplotype of *B. truncatus*; USCH, urogenital schistosomiasis

### Analysis of sequence data

After alignment and editing, the query length of the *cox*1 sequences was 607 bp. The query sequences for *B. globosus* were highly similar (> 98.0%) to *B. globosus* from Pietermaritzburg, South Africa (GenBank: AM286290 and AM286289). The *B. truncatus* query sequences had a high similarity (99.34%) to *B. truncatus* (GenBank: MG759464) from Zimbabwe.

### Haplotype and population genetic diversity, and genetic divergence of *B. globosus*

Twelve unique *B. globosus cox*1 haplotypes were found from the 166 samples sequenced. The overall haplotype diversity exceeded 0.5 with a nucleotide diversity estimate of 0.002. The haplotypes were not highly divergent (pairwise distance of 0.002–0.007). Within the sites and within communities as expected, haplotype diversity was generally low but ranging between 0–0.786 (Table 2). The percentage occurrence of each haplotype differed among sites and communities. One common haplotype (H1) was found in all but one locality representing 114 (68.7%) of the 166 samples analysed. Some haplotypes were locally restricted being observed in one site only but for those represented by a considerable number of snails, there was a clear pattern of haplotype sharing within the river systems with high haplotype sharing being observed in sites that are close to each other or within the same river (Table 1). In Mupfure River, which had the highest number of haplotypes among the rivers, sites that were close together shared the same haplotypes (Tables 1, 2). Some sites had complete *B. globosus* homogeneity and this was observed mainly in Kamoyo stream where all sites had only one haplotype (H1). The homogeneity in Nduna/Kamoyo stream was also supported by lack of haplotype diversity. However, among the sites that had more than one haplotype, overall intra-site haplotype diversity estimates ranged between 0.250–0.786 and a considerable number of sites recorded a haplotype diversity of more than 0.5. The maximum number of haplotypes that could be found per site did not exceed four. The mean overall diversity for *B. globosus* was 0.002.

**Table 2:** Mitochondrial cytochrome *c* oxidase 1 diversity of *B. globosus* and *B. truncatus* collected from the Madziwa area, Zimbabwe

Species	Location	*n*	H	Hd ± SD	π	θ	Tajima’s D statistic	Fu’s Fs statistic
*B. globosus*	Overall	166	12	0.514 ± 0.046	0.002	0.008	− 2.136*	− 3.604
	Chihuri C	32	6	0.637 ± 0.076	0.003	0.009	− 2.307**	0.397
	Site 1	8	2	0.250 ± 0.180	0.000	0.001	− 1.055	− 0.182
	Site 2	8	3	0.464 ± 0.200	0.001	0.001	− 1.310	− 0.999
	Site 3	8	2	0.536 ± 0.123	0.001	0.001	1.167	0.866
	Site 4	8	4	0.786 ± 0.113	0.010	0.014	− 1.466	2.726
	Kaziro C	19	3	0.374 ± 0.130	0.001	0.001	− 0.729	− 0.671
	Site 7	7	1	0	0	–	–	–
	Site 8	4	2	0.500 ± 0.265	0.001	0.001	− 0.612	0.172
	Site 9	8	3	0.607 ± 0.164	0.001	0.001	− 0.448	− 0.478
	Mupfure C	98	10	0.558 ± 0.057	0.002	0.009	− 2.202**	− 2.108
	Site 5	10	3	0.644 ± 0.101	0.002	0.002	− 0.356	0.390
	Site 6	9	4	0.583 ± 0.183	0.001	0.002	− 1.610	− 1.283
	Site 10	8	3	0.714 ± 0.123	0.014	0.013	0.675	6.022
	Site 11	8	3	0.679 ± 0.122	0.001	0.001	0.069	− 0.224
	Site 12	7	2	0.476 ± 0.171	0.001	0.001	0.559	0.589
	Site 13	8	4	0.643 ± 0.184	0.001	0.002	− 1.448	− 1.832
	Site 14	8	2	0.250 ± 0.180	0.000	0.001	− 1.055	− 0.182
	Site 15	8	3	0.607 ± 0.164	0.001	0.001	− 0.448	− 0.478
	Site 16	8	3	0.464 ± 0.200	0.001	0.002	− 1.448	− 0.305
	Site 17	8	2	0.250 ± 0.180	0.000	0.001	− 1.055	− 0.182
	Site 18	8	2	0.571 ± 0.094	0.001	0.001	1.444	0.966
	Site 19	8	2	0.250 ± 0.180	0.000	0.001	− 1.055	− 0.182
	Nduna C	17	1	0	0	–	–	–
	Site 20	4	1	0	0	–	–	–
	Site 21	5	1	0	0	–	–	–
	Site 22	8	1	0	0	–	–	–
*B. truncatus*	Overall	16	3	0.575 ± 0.080	0.017	0.012	1.617	11.547
	Nyamaruru R	1	1	0	0	–	–	–
	Mupfure R	7	3	0.286 ± 0.196	0.005	0.007	− 1.610*	4.273
	Kamoyo S	8	1	0	0	–	–	–

*Abbreviations*: n, number of sequences, h, number of unique haplotypes per site; Hd, haplotype diversity; π, nucleotide diversity; θ, theta per site; C, Community; R, River; S, Stream; SD, standard deviation

*P < 0.05, **P < 0.01

Nucleotide divergence between and within communities was low ranging between 0–0.003 (Table 3). Expectedly, the genetic divergence between *B. globosus* and *B. truncatus* was high (*Fst* = 0.920) supporting the species delimitation. When the pairwise fixation index (*Fst*) among the sites was estimated between the populations, a significant number of the populations showed significant levels of differentiation from each other whilst others showed no genetic differentiation. The pairwise *Fst* values ranged between 0–0.429 for *B. globosus.* A high genetic differentiation of > 30% and above was mainly found among different river systems demonstrating geographically defined gene population structuring (see pairwise *Fst* values for *B. globosus* in Additional file 1: Table S2). This suggests that the river system maybe acting as a barrier to outbreeding. However, genetic differentiation was very low, ranging between 0.004–0.061. Within the communities and river systems, evolutionary divergence ranged between 0–0.003 (Table 4).

**Table 3:** Nucleotide divergences among *B. globosus* populations in different communities

Community	Mupfure	Kaziro	Chihuri	Nduna
Mupfure	0.002			
Kaziro	0.002	0.001		
Chihuri	0.003	0.002	0.003	
Nduna	0.001	0.0003	0.002	0.000

**Table 4:** Estimation of evolutionary divergence within localities

Locality	Mean distance ± SD
*B. globosus* overall	0.002 ± 0.000
Mupfure community	0.002 ± 0.001
Kaziro community	0.001 ± 0.001
Chihuri community	0.003 ± 0.001
Nduna community	0.000
*B. truncatus* overall	0.017 ± 0.003
Mupfure River	0.017 ± 0.004
Kamoyo Stream	0.000
Overall mean distance	0.022 ± 0.003

*Abbreviation*: SD, standard deviation

### Haplotype, population genetic diversity and genetic divergence of *B. truncatus*

*Bulinus truncatus* populations were lower in number compared to *B. globosus* and they were only found in the Nyamaruru, Mupfure rivers and Kamoyo stream. Out of the 16 samples of *B. truncatus* analysed there were three unique *cox*1 haplotypes. The overall haplotype diversity was 0.575 with an estimated nucleotide diversity of 0.017. The average haplotype diversity for *B. truncatus* was also 0.017.

Within the river systems, haplotype diversity ranged between 0–0.286 (Table 2). All the three haplotypes were recorded in Mupfure River while the other two rivers exhibited a complete homogeneity (Table 2). The mean pairwise divergence among the haplotypes ranged from 0.017 to 0.034. The pairwise *Fst* value of *B. truncatus* was generated only for Mupfure River and Kamoyo stream, which had more than one sample (0.922). Nucleotide divergence between the Mupfure River and Kamoyo stream was 0.031 (Table 3).

### Neutrality tests

Tajima’s D statistic and Fu’s Fs statistic showed no selection among the *B. globosus* populations among the sites. However, overall there were significant negative values for both Tajima’s D statistic and Fu’s Fs statistic. In the case of *B. truncatus*, overall, both neutrality tests were not significant, but Tajima’s D was significant for Mupfure River (Table 2).

### Phylogenetic structuring

#### Bulinus globosus

**Fig. 2:**
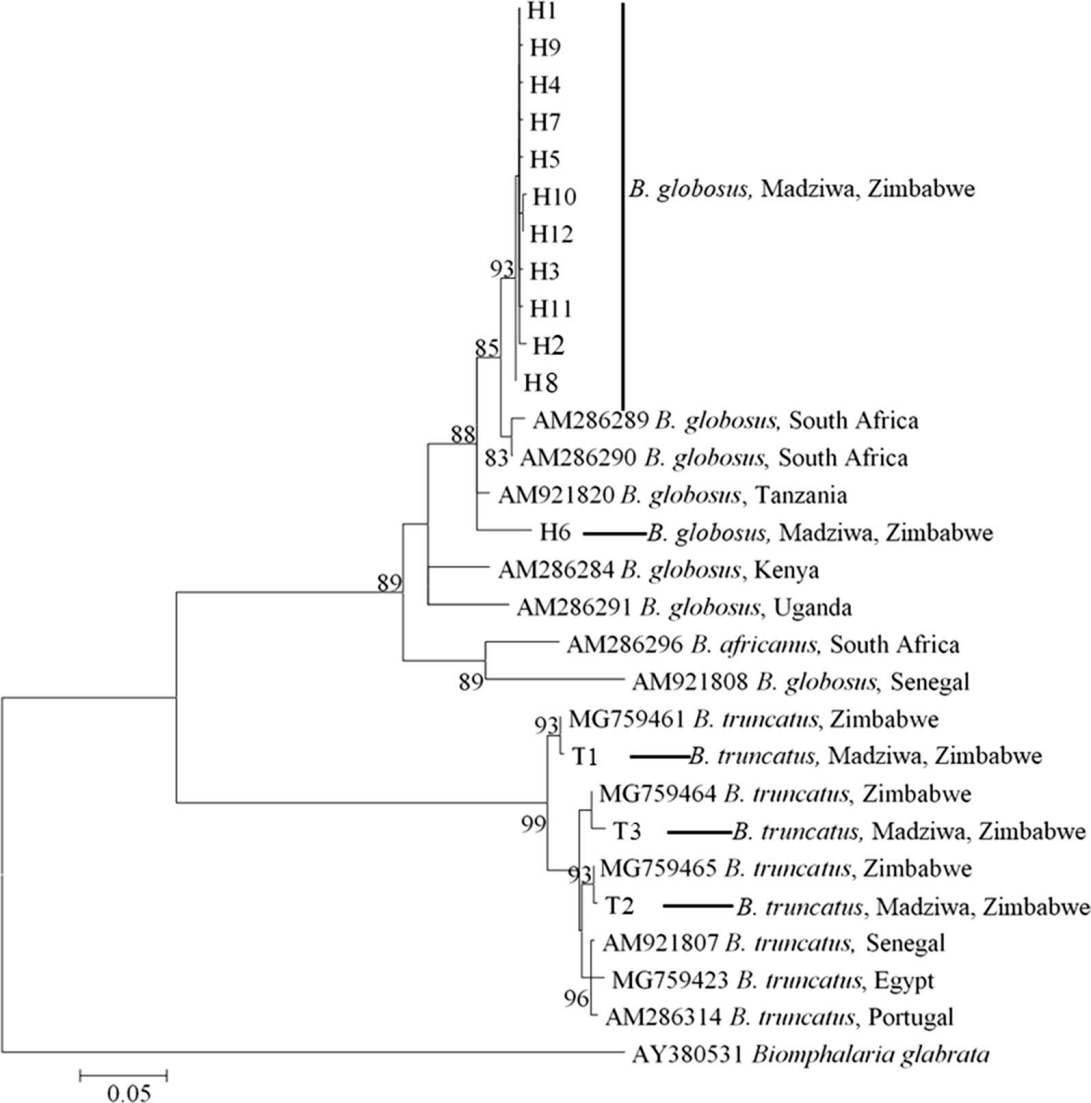
Maximum likelihood phylogenetic analysis of the *B. globosus* and *B. truncatus* haplotypes collected in Madziwa, Zimbabwe, in relation to other *Bulinus* species. Each terminal bar is marked using the species name and the code given to the haplotype, e.g. H1 or T1. *Bulinus globosus* and *B. truncatus* haplotypes are represented by the letter H and T, respectively. The bootstrap values below 80% are not shown. There were a total of 607 positions in the final dataset. The accession numbers of the individual sequences forming the haplotypes are shown in Additional file 1: Table S3. The distribution of sequences among *B. globosus* haplotypes was as follows: haplotype 1 (*n* = 114 samples); haplotype 2 (*n* = 14); haplotype 3 (*n* = 11); haplotype 4 (*n* = 9); haplotype 5 (*n* = 8); haplotype 6 (*n* = 3); haplotype 8 (*n* = 2); haplotypes 7, 9, 10, 11 and 12 (*n* = 1 sample each). For *B. truncatus*, there were only three haplotypes. Among these three, the most common haplotype (T1) represented 10 samples, T2 representing 5 samples and T3 representing 1 sample

**Fig. 3:**
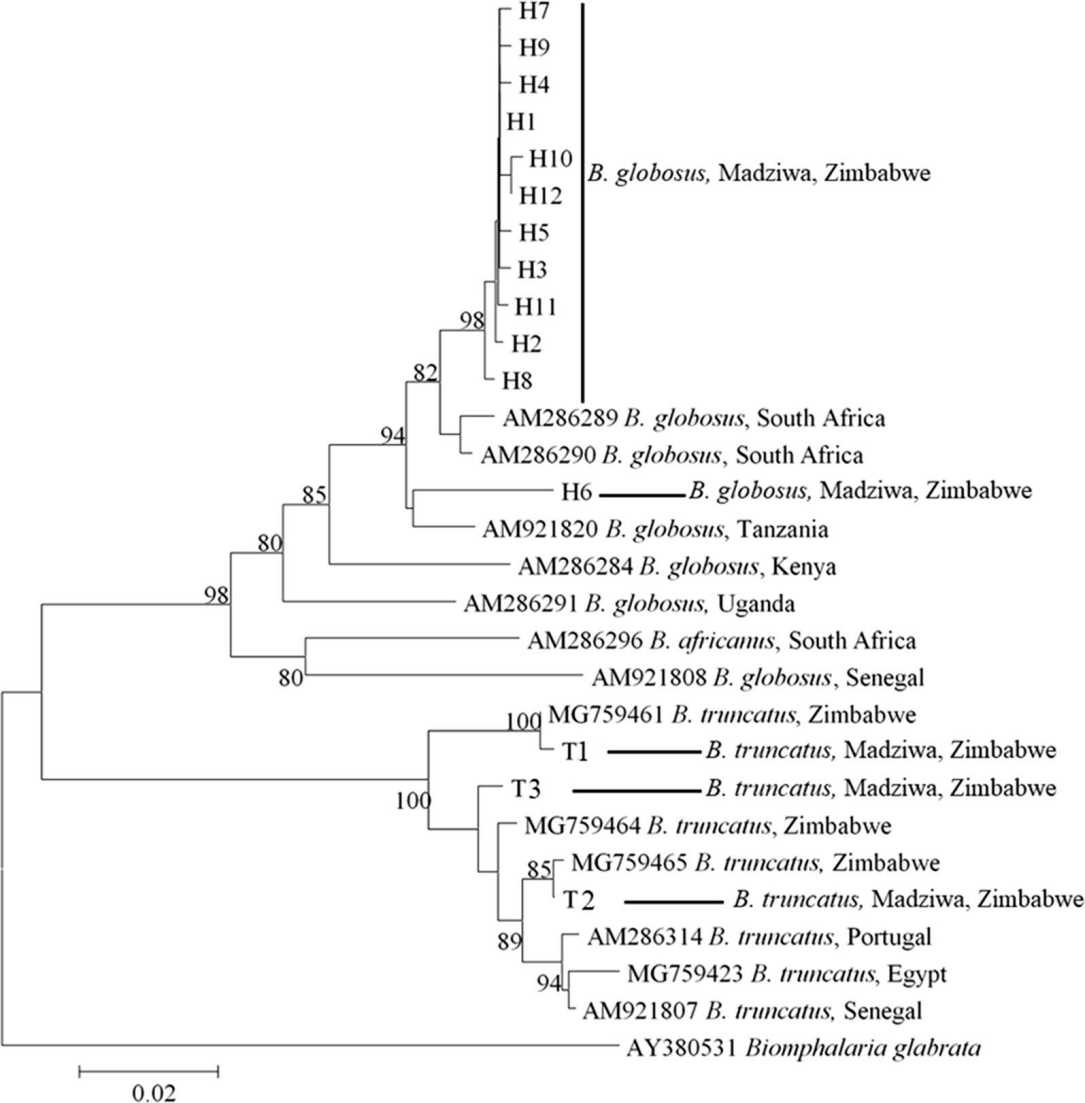
Neighbor-joining phylogenetic tree of the *B. globosus* and *B. truncatus* haplotypes collected in Madziwa, Zimbabwe, in relation to other *Bulinus* species. Each terminal bar is marked using the species name and the code given to the haplotype, e.g. H1 or T1. *Bulinus globosus* and *B. truncatus* haplotypes are represented by the letter H and T, respectively. The bootstrap values below 80% are not shown. The accession numbers of the individual sequences forming the haplotypes are shown in Additional file 1: Table S3

**Fig. 4:**
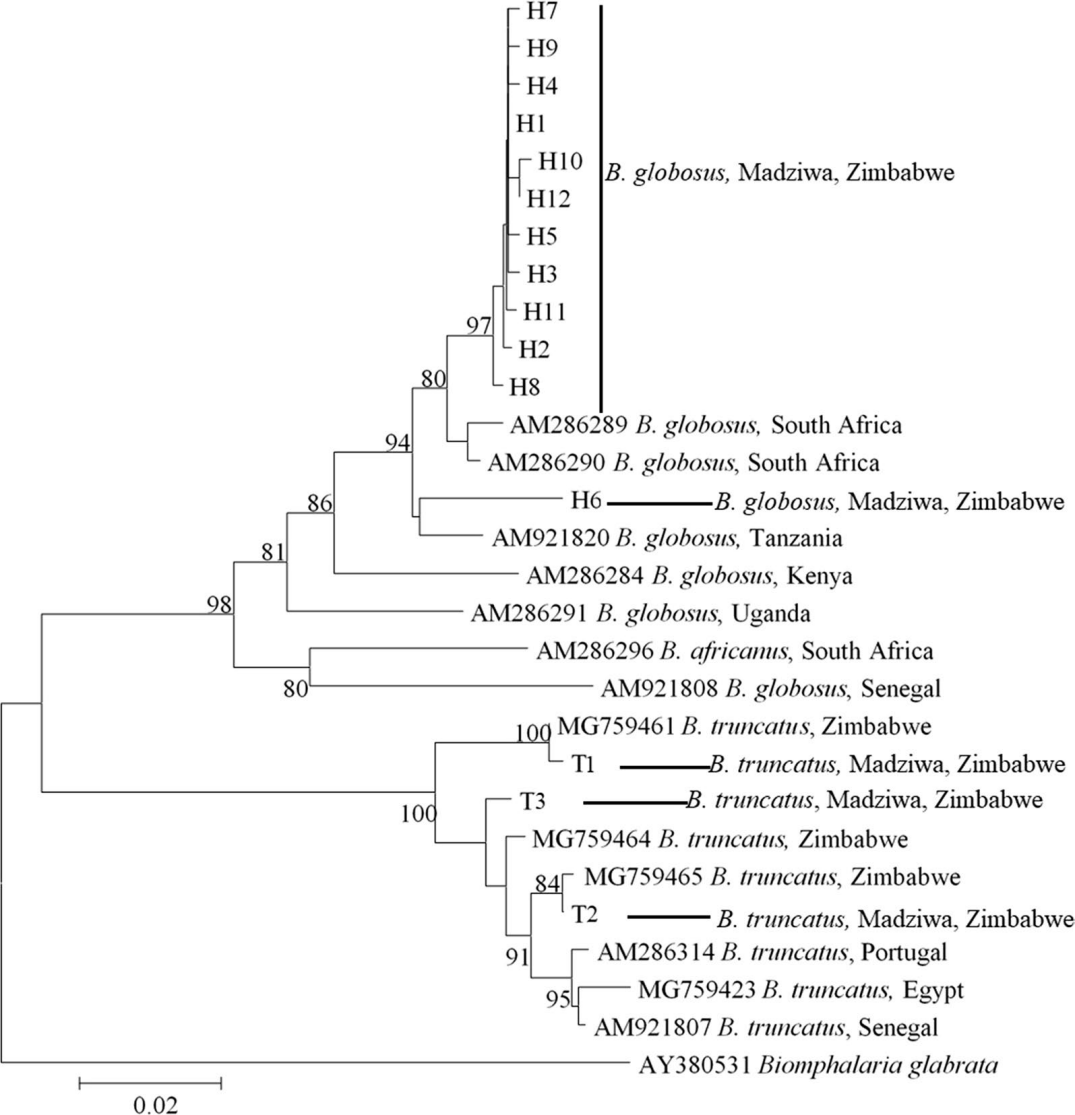
Minimum evolution phylogenetic tree of the *B. globosus* and *B. truncatus* haplotypes collected in Madziwa, Zimbabwe, in relation to other *Bulinus* species. Each terminal bar is marked using the species name and the code given to the haplotype, e.g. H1 or T1. *Bulinus globosus* and *B. truncatus* haplotypes are represented by the letter H and T, respectively. The significant bootstrap values for 1000 replicates are shown next to the branches. The accession numbers of the individual sequences forming the haplotypes are shown in Additional file 1: Table S3

**Fig. 5:**
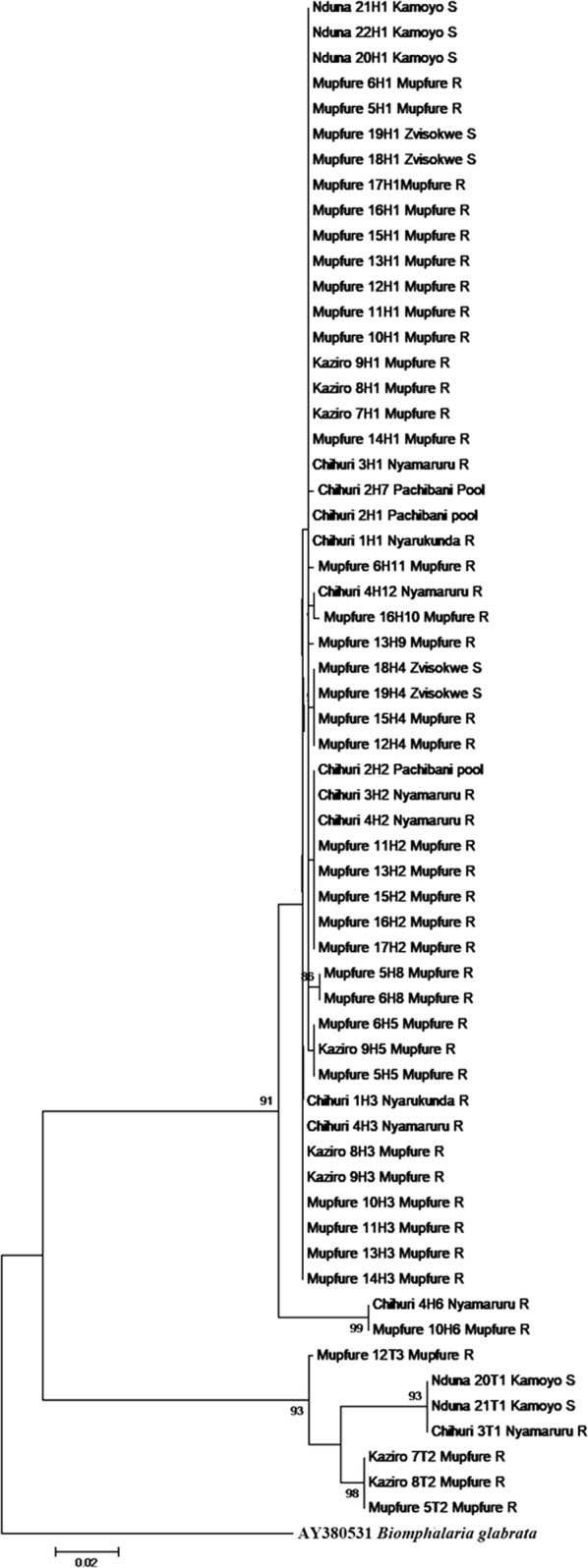
Detailed Maximum Likelihood phylogenetic tree of *B. globosus* and *B. truncatus* showing distribution of haplotypes by site, community and water system. Each terminal bar is marked using the species name, site number and the code given to the haplotype, e.g. H1 or T1. *Bulinus globosus* and *B. truncatus* haplotypes are represented by the letter H and T, respectively. The significant bootstrap values for 1000 replicates are shown next to the branches. The accession numbers of the individual sequences forming the haplotypes are shown in Additional file 1: Table S3

Eleven of the 12 *B. globosus* haplotypes clustered closely with *B. globosus* from South Africa. H8 also had strong support for divergence away from the main haplotype group. One haplotype (H6) was an outlier and clustered more closely with a sample from Tanzania (Figs. 2, 3, 4). The topology of the three phylogenetic trees (Figs 2, 3, 4; ML, NJ and ME, respectively) was the same. H6 was found in two rivers (Nyamaruru and Mupfure), while H8 occurred in Mupfure River only. H4 was present in Mupfure River and Zvisokwe, a small stream branching into Mupfure River (Fig. 5). These water systems are also mapped in Fig. 1. The accession numbers corresponding to each sequence for all the haplotypes are found in Additional file 1: Table S3.

#### *Bulinus truncatus*

The three *B. truncatus* haplotypes clustered with data from previously characterized samples from Zimbabwe, but there was a considerable phylogenetic separation between the groups (Figs. 2, 3, 4). The topology of the three phylogenetic trees (Figs. 2, 3, 4; ML, NJ and ME, respectively) was the same. There was a strong geographical clustering as the clusters were made up of haplotypes from localities that were close together or from a single locality (Fig. 5). While T2 and T3 were found in Mupfure River, T1 was present in Kamoyo stream and Nyamaruru River. The accession numbers corresponding to each sequence for all the haplotypes are found in Additional file 1: Table S3.

## Discussion

The present results show that *Bulinus* population diversity exists in Madziwa, Zimbabwe. While the shell microsculpture clearly differed between the two species, as previously observed by Brown [8], identification can be confusing due to the twisting of the columella in *B. truncatus* and *B. africanus*-group. Within the *B. africanus*-group, it is difficult to clearly distinguish between *B. africanus* and *B. globosus* because the main morphologically variable feature, the penis, varies in size and shape due to parasitic infection, thus was not used in the present study to differentiate the two species. Likewise, as corroborated by Brown [8], it was difficult to construct a dichotomous key within the *B. tropicus/truncatus* group because of the continuous variation in shell shape between the species in this group.

Amplification of the *cox*1 shows that, within the species morphologically identified as *B. globosus* and *B. truncatus*, complete homogeneity for both was only recorded in Kamoyo stream, Nduna. In other sites, on average within the same site, approximately 25% of the samples had a different haplotype. Although the diversity recorded is low, it is expected within species. However, the diversity recorded in this study is greater than that recorded in Cameroon by Nalugwa et al. [36] in *B. truncatus* and *B. forskalii* populations where on average 16% of the snails sampled had a different haplotype. The ability of *Bulinus* spp. to self-fertilize and cross-breed induces some genetic consequences [37]. *Bulinus globosus* preferentially outbreeds increasing its diversity, therefore, higher genetic variability in the species is expected compared to that of *B. truncatus*, which preferentially self-fertilizes [38]. However, a previous study has shown that diversification is high in polyploid species such as *B. truncatus* [39].

Except for the dominant *B. globosus cox*1 haplotype, which spanned across almost all the sites where *B. globosus* was found, the diversity in haplotypes among the sites showed that genetic drift most likely due to bottleneck effects is a probable factor in determining diversity within and between species in this area. Our results showed homogeneity within the *B. globosus* originating from Nduna, Kamoyo stream, compared to the other communities. The complete homogeneity could be a result of natural selection, drift or perhaps a recent introduction of the intermediate host snail into these sites. Nevertheless, as presented by Schmid-hempel & Stauffer [39], host-parasite susceptibility can increase with loss of genetic variation; the snails in this river had the highest infection rates compared to the other rivers regardless of the fact that very few sites were sampled. On the contrary, a schistosomiasis prevalence study in the area has shown that Nduna community has a significantly low prevalence of the disease compared to Chihuri and Mupfure communities [40]. Both Mupfure and Chihuri had a higher genetic diversity of the intermediate host as compared to Nduna. Furthermore, the two areas had some infected snails at some point during the year. Thus, the contradicting scenario might be explained by other factors related to water contact behaviour of the human communities, given that schistosomiasis infection is a function of duration of exposure [41]. We may also hypothesize that the transmission of the disease in Nduna started relatively recently compared to other communities due to the absence of the intermediate host snail. Alternatively, it is highly probable that there is a risk of occurrence of schistosomiasis-transmitting genotypes becoming widely successful with selection of traits independent of parasitic infection [42]. The expanding population theory is corroborated by the significant test of neutrality for both Tajima’s D statistic and Fu’s Fs statistic for the whole *B. globosus* population or the *B. truncatus* population in Mupfure River. Similar results have also been reported by Zein-Eddine et al. [13].

Samples that were collected and analysed in this study, were from sites within close proximity to one another and they had an overall low genetic variation contrary to expectations due to limited gene flow between the populations. However, reproductive isolation, due to the different river systems, may have allowed independent evolution and divergence of these populations leading to formation of different haplotypes. The reproductive isolation might result in complete homogeneity in Kamoyo stream and the presence of ‘unique’ haplotypes in some of the rivers. Like in the previous study by Standley et al. [43] for *Biomphalaria choanomphala* in Lake Victoria where the ‘unique’ haplotypes were locally restricted to the central and western regions of the lakeshore in Uganda, in the present study, the ‘unique’ haplotypes are also restricted to Nyamaruru and Mupfure rivers.

To our knowledge, the present study provides the first genetic assessment of combined data for *B. globosus* and *B. truncatus* from Zimbabwe, where urogenital schistosomiasis is endemic. However, more sophisticated statistical analyses of these haplotypes should be performed to examine the levels of divergence which may lead to species delimitation.

## Conclusions

A detailed understanding of genetic variability within intermediate host snail species is important for understanding disease epidemiology. A novel insight from our study is the mitochondrial *cox*1 variability within *B. globosus* and additional *B. truncatus* haplotypes in Madziwa area, Zimbabwe. Further investigations using new tools for detection of infection will help investigate the snail populations involved in transmission. The identification of *Bulinus* in these areas will support targeted control efforts for schistosomiasis in the study area. Further studies over a large spatial area with higher sample size are also required to understand the genetic demographic patterns of these intermediate host snail species.

## Supplementary information

**Additional file 1: Table S1.** List of sequences drawn from the Genbank incorporated for phylogenetic analysis. **Table S2.** Fst values for *B. globosus* populations. **Table S3.** Accession numbers of individual sequences for the haplotypes recorded in Madziwa, Zimbabwe.

## Data Availability

Data supporting the conclusions of this article are included within the article and its additional files. The newly generated sequences were submitted to the GenBank database under the accession numbers MN397785–MN397822. The datasets analysed during the present study are available from the corresponding author upon reasonable request.

## Abbreviation

*cox*1: cytochrome oxidase subunit 1
